# Use of Chronic Kidney Disease to Enhance Prediction of Cardiovascular Risk in Those at Medium Risk

**DOI:** 10.1371/journal.pone.0141344

**Published:** 2015-10-23

**Authors:** Yook Chin Chia, Hooi Min Lim, Siew Mooi Ching

**Affiliations:** 1 Department of Primary Care Medicine, University of Malaya Primary Care Research Group (UMPCRG), Faculty of Medicine, University of Malaya, Kuala Lumpur, Malaysia; 2 Department of Family Medicine, Faculty of Medicine and Health Sciences, Universiti Putra Malaysia, Serdang, Malaysia; 3 Malaysian Research Institute on Ageing, Faculty of Medicine and Health Sciences, Universiti Putra Malaysia, Serdang, Malaysia; The University of Tokyo, JAPAN

## Abstract

Based on global cardiovascular (CV) risk assessment for example using the Framingham risk score, it is recommended that those with high risk should be treated and those with low risk should not be treated. The recommendation for those of medium risk is less clear and uncertain. We aimed to determine whether factoring in chronic kidney disease (CKD) will improve CV risk prediction in those with medium risk. This is a 10-year retrospective cohort study of 905 subjects in a primary care clinic setting. Baseline CV risk profile and serum creatinine in 1998 were captured from patients record. Framingham general cardiovascular disease risk score (FRS) for each patient was computed. All cardiovascular disease (CVD) events from 1998–2007 were captured. Overall, patients with CKD had higher FRS risk score (25.9% vs 20%, p = 0.001) and more CVD events (22.3% vs 11.9%, p = 0.002) over a 10-year period compared to patients without CKD. In patients with medium CV risk, there was no significant difference in the FRS score among those with and without CKD (14.4% vs 14.6%, p = 0.84) However, in this same medium risk group, patients with CKD had more CV events compared to those without CKD (26.7% vs 6.6%, p = 0.005). This is in contrast to patients in the low and high risk group where there was no difference in CVD events whether these patients had or did not have CKD. There were more CV events in the Framingham medium risk group when they also had CKD compared those in the same risk group without CKD. Hence factoring in CKD for those with medium risk helps to further stratify and identify those who are actually at greater risk, when treatment may be more likely to be indicated.

## Introduction

The prevalence of chronic kidney diease (CKD) is increasing in Asian countries and is now even higher than the developed western countries. [1–4] CKD is associated with atherosclerosis in coronary, cerebral and peripheral arterial circulation and hence is an independent risk factor for cardiovascular disease (CVD). [5–9] Patients with CKD developed significant CVD morbidity and mortality before they even reach end-stage renal disease (ESRD). [10]

Cardiovascular (CV) risk stratification is important in identifiying those with high global CV risk so that primary prevention and treatment can be initiated early to reduce CV events. [11] Several CV risk prediction models have been developed to estimate risk, among them is the Framingham risk score (FRS). The FRS is the most widely studied and has been validated in multiple populations and found to perform well in predicting the 10-year CV risk. [12–14] Based on this risk stratification model, it is recommended that those with high risk be treated and those with low risk should not be given any treatment. The recommendation for those medium risk is less clear and uncertain. Hence we aimed to assess whether the presence or absence of CKD will improve the prediction in those with medium risk.

## Materials and Methods

This is a 10-year retrospective cohort study. The study is conducted in an outpatient primary care clinic at University Malaya Medical Centre (UMMC), a teaching hospital in the Klang valley of Kuala Lumpur Malaysia. Subjects were randomly selected from the patients registered with the clinic. Ethnics approval was obtained from the Ethnics Committee of University Malaya Medical Centre. As this was a retrospective study based on patient records, and as all data entry, analysis and results output was anonymized, no informed consent, verbal or written was obtained. Ethics approval for our study, based on this study design and methodology was obtained and granted by the Ethics Committee of our institution. (University of Malaya Medical Centre Ethics Committee/IRB Reference Number 691.1).

### Inclusion and exclusion criteria

All adults aged 30 years and older, without any cardiovascular event at baseline (1998), with complete variables for Framingham general CVD risk score calculation and serum creatinine level for calculation of estimated glomerular filtration rate (eGFR) were selected into the analysis.

We excluded patients with incomplete variables for Framingham risk score calculation at baseline, those with missing data on serum creatinine at baseline or those subjects with missing data on CVD event in 2007.

### Data collection

Subjects were randomly selected using a computer-generated number based on patients’ clinic registration numbers. All sociodemographic data, variables for risk score calculation and variables for eGFR calculation were extracted from paper-based medical records manually. Use of antihypertensive medications, hypoglycaemic agents and lipid-lowering agents were captured as well.

Framingham general CVD risk score for each subject was calculated using age, systolic blood pressure (BP) (treated or not treated), total cholesterol level, high-density lipoprotein (HDL), cholesterol level, smoking and diabetes mellitus (DM). [15] 10-year CVD risk for each individual was categorised into low risk (<10%), moderate risk (10–20%) and high risk (>20%).

CVD events were captured from patients’ medical records. These included fatal and non-fatal cardiovascular heart disease, fatal and non-fatal stroke, heart failure and peripheral vascular disease were captured from the medical records. For those subjects who did not complete their 10-year follow-up in our clinic, we traced their hospital records to ascertain their CV status. For those who had died, we captured the cause of death from the patient’s hospital records where a diagnosis of the cause of death was made. If the cause of death was a CV event, we included and counted this as a CV event. For those who did not continue to be seen at our hospital, we called the patient or the family to ascertain the patient’s status. Some were well without any CV events but decided to be followed up in a clinic closer to their own home town where they were living. For those who had died, we conducted a verbal autopsy and ascertained the cause of death as certified by the attending doctor at their home town. Again, if the cause of death was a CV event, we included and counted this as a CV event that had occured.

Patients with non-fatal cardiovascular heart disease refer to those who suffered from a myocardial infarction diagnosed by clinical features, electrocardiographic (ECG) findings and elevated biochemical markers of myocardial necrosis but who survived. Fatal cardiovascular heart disease included by myocardial infarction or its complications like ventricular tachycardia and which resulted in death of the patient. Angina was diagnosed clinically by the attending doctors supported by relevent diagnostic investigations. Stroke was defined according to the clinical diagnosis made by the attending clinician and supported by imaging. Heart failure referred to the diagnosis made by attending doctors based on clinical features and echocardiograpic findings.

All blood tests were performed by the chemical laboratory in the hospital which is certified by the Royal College of Pathologists of Australasia standards. Serum creatinine levels were captured and were used for calculation of eGFR: according to the Chronic Kidney Disease Epidermiology Collaboration Equation (CKD-EPI). [16] Chronic kidney disease was defined as eGFR <60ml/min/1.73m2 in accordance to the staging by KDIGO.[17]

Blood pressure was measured by the attending doctors using mercury sphygmomanometer during routine clinical practice. Hypertension is defined according to the JNC guidelines.[18] Use of antihypertensive agents were captures as well. DM is defined as documented by attending doctor or the use of hypoglycaemic agents or both. Smoker is defined as if they were still smoking currently. Non-smokers were those who never smoke or currently not smoking. Total cholesterol, triglyceride, HDL and low-density lipoprotein (LDL) cholesterol levels were also collected.

### Statistical analysis

All statistical analysis was carried out using Statistical Package for Social Sciences (SPSS V.21). Continuous data are described with mean and stardard deviation if the distribution is normal; median and interquartile range (IQR) if the distribution is skewed. Chi-square test was used for the analysis of categorical variables. Student T-test was used for the comparison of means if it is normally distributed. P-value of <0.05 is considered significant.

## Results

**Fig 1:**
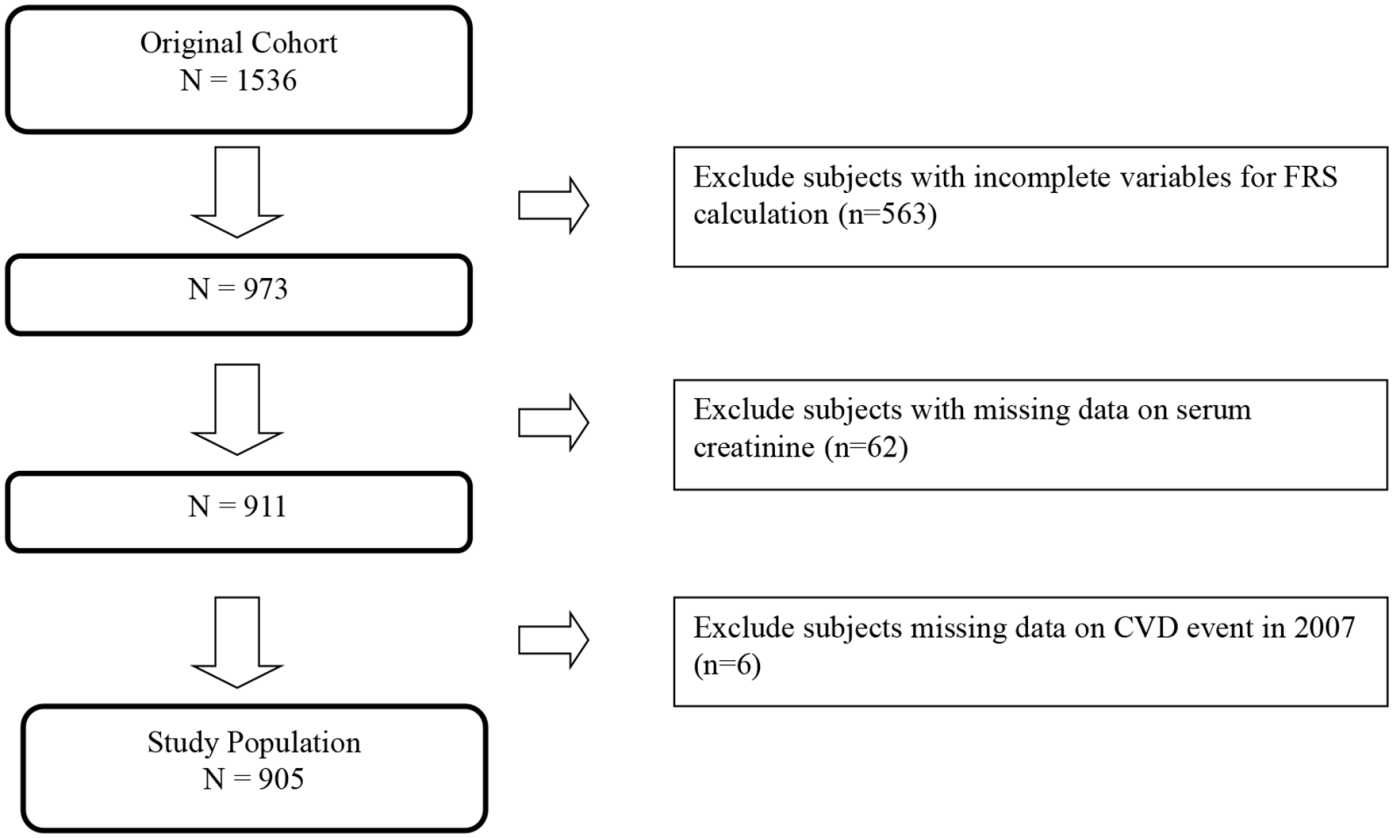
Flowchart of patients included in this study.

There were 1536 patients in our original cohort study. We excluded 563 patients who did not have all the variables to calculate the Framingham general CVD risk score. These excluded patients were found to have no difference in their clinical risk factors profile compared to those included in our analysis. [12] Out of 973 patients, we further excluded 62 patients who did not have the serum creatinine to calculate eGFR. Another 6 patients were excluded because we could not ascertain their CVD status at the end of 10 years. Thus, a total of 905 subjected were eligible for analysis of this study. (Fig 1)

Table 1 shows the sociodemographic data and CV risk factors of the study population at baseline (1998). The mean age of the subjects was 57 years and the major ethnic group was Chinese (45.5%). More than half of the subjects (60.6%) have hypertension and nearly half were diabetics (47.8%) but smoking was low (5.7%). The use of statin was very low (9.3%). The total 10-year CVD events was 120 (13.3%) in year 2007. The overall FRS risk score is high (21.5%) but the event rate was lower (13.3%). The events included 2 fatal and 6 non-fatal myocardial infarction, 72 angina, 4 fatal and 34 nonfatal stroke. Seven subjects had heart failure.

**Table 1:** Sociodemographic data and cardiovascular risk factors of the study population in year 1998 (n = 905).

**Age, year (mean±SD)**	**57.08 ±9.9**
**Female (n,%)**	**594 (65.6)**
**Race (n,%)**	
**Malay**	**208 (23.0)**
**Chinese**	**412 (45.5)**
**Indian**	**272 (30.1)**
**Others**	**11 (1.2)**
**Weight, kg (mean±SD)**	**65.7 ±12.9**
**Smoking (n,%)**	**52 (5.7)**
**Systolic BP, mmHg (mean±SD)**	**140.9 ±18.5**
**Diastolic BP, mmHg (mean±SD)**	**84.8 ±10.0**
**Hypertension (n,%)**	**548 (60.6)**
**Types of antihypertensive agents used(n,%)**	
**ACE-i/ARB**	**68 (7.5)**
**B—blockers**	**288 (31.8)**
**CCB**	**239 (26.4)**
**Diuretics**	**61 (6.7)**
**α-blockers**	**35 (3.9)**
**Diabetes mellitus (n,%)**	**433 (47.8)**
**Types of diabetic medications used(n,%)**	
**Metformin**	**208 (23.0)**
**Sulphonyurea**	**325 (35.9)**
**Insulin**	**8 (0.9)**
**Total cholesterol, mmol/L (mean±SD)**	**6.1 ±1.9**
**LDL cholesterol, mmol/L (mean±SD)**	**3.7 ±1.0**
**HDL cholesterol, mmol/L (mean±SD)**	**1.2 ±0.4**
**Use of statins (n,%)**	**84 (9.3)**
**Serum creatinine, μmol/L (mean±SD)**	**80.3 ±24.7**
**eGFR, mL/min/1.73m** ^**2**^ **(mean±SD)**	**81.5 ±18.9**
**FRS in 1998, % (median, IQR)**	**21.5 (13.2–30.0)**
**Total CVD events in 2007 (n,%)**	**120 (13.3%)**

CV cardiovascular; kg kilogram; BP blood pressure; ACE-i Angiotensin-converting enzyme inhibitor; ARB angiotensin receptor blocker; CCB calcium channel blocker; eGFR estimated glomerular filtration rate; CVD cardiovascular disease; FRS Framingham general CVD risk score

Table 2 shows the mean FRS score according to CVD risk category and CKD status. Table 3 compares Framingham general CV risk score and CVD events rate among patients with and without CKD. Although CKD is not one of the variable in the FRS score calculation, patients with CKD had higher FRS risk score (25.9% vs 20%, p = 0.001.%). Additionally, CKD patients had more CVD events in 10-year period compared to those without CKD (22.3% vs 11.9%, p = 0.002). When we analysed the characteristics of CKD patients, we noticed that the major factors for CKD were older age and hypertension.

**Table 2:** Comparison of mean Framingham Risk Score according to CVD risk category and CKD status (N = 905).

FRS Risk Category	Mean FRS score (%)		p-value
	CKD	No CKD	
**Low risk, mean score (6.4%)**	**4.7**	**6.5**	**0.07**
**Medium risk, mean (14.5%)**	**14.4**	**14.6**	**0.84**
**High risk, mean (27.8%)**	**28.5**	**27.6**	**0.006**
**Total, mean**	**25.9**	**20**	**0.001**

FRS = Framingham General CV risk score; CKD = chronic kidney disease

**Table 3:** Comparison of the Framingham General CV risk category, chronic kidney disease status and cardiovascular disease event over 10 year (n = 905).

FRS Risk Category	Presence of CKD	N (%)	CVD event rate,%	p-value
**Low risk (<10%)**	**CKD**	**4/129 (3.1)**	**0% (0/4)**	**0.60**
	**No CKD**	**125/129 (96.9)**	**6.4% (8/125)**	
**Medium risk (10–20%)**	**CKD**	**15/271 (5.5)**	**26.7% (4/15)**	**0.005**
	**No CKD**	**256/271 (94.5)**	**6.6% (17/256)**	
**High risk (>20%)**	**CKD**	**102/505 (20.2)**	**22.5% (23/102)**	**0.18**
	**No CKD**	**403/505 (79.8)**	**16.5% (68/403)**	
**Total (n = 905)**	**CKD**	**121/905 (13.4)**	**22.3% (27/121)**	**0.002**
	**No CKD**	**784/905 (86.6)**	**11.9% (93/784)**	

FRS = Framingham General CV risk score; CKD = chronic kidney disease

For patients with medium CV risk, there was no significant difference in the FRS among those with and without CKD (14.4% vs 14.6%, p = 0.84) (Table 2). However, in this medium risk group, patients with CKD had higher CV events compared to those without CKD (26.7% vs 6.6%, p = 0.005) (Table 3). For those in medium risk group without CKD, their CV event was much lower than predicted. This was in spite of the fact that there was no difference in the traditional CV risk factors profile between CKD and non-CKD patients in medium risk group (Table 4). For low and high risk groups, the presence or absence of CKD did not make any difference in term of CV risk prediction.

**Table 4:** Comparison of cardiovascular risk factors profile between patients with and without chronic kidney disease in the medium risk group.

CV risk factors profile	CKD (n = 15)	No CKD (n = 256)	p-value
**Age, year (mean±SD)**	**60 ±11.3**	**53.2 ±8.8**	**0.005**
**Sex, female, n (%)**	**14 (93.3%)**	**205 (80.1%)**	**0.21**
**Hypertension, n (%)**	**7 (46.7%)**	**133 (52%)**	**0.69**
**Systolic BP, mmHg (mean±SD)**	**130 ±16.0**	**137 ±16.1**	**0.12**
**Diabetes mellitus, n (%)**	**4 (26.7)**	**104 (40.6)**	**0.28**
**Total cholesterol, mmol/l (mean±SD)**	**5.96 ±1.67**	**6.07 ±1.01**	**0.69**
**LDL cholesterol, mmol/l (mean±SD)**	**3.25 ±0.82**	**3.84 ±1.15**	**0.39**
**Smoking, n (%)**	**0**	**5 (2%)**	**0.60**

CV cardiovascular; CKD chronic kidney disease; CVD cardiovascular disease; LDL low-density lipoprotein

Table 5 shows the treatment profiles by CVD risk category and CKD status of the study population. Overall more of those in the high risk group received treatment than those in the medium and low risk groups regardless of CKD status. However in those patients who were of medium CV risk, those with CKD were receiving less treatment across all medications compared to those without CKD.

**Table 5:** Treatment profiles by CVD risk category and CKD status (n = 905).

FRS Risk Category	Number of patients on anti-hypertensive agents (n,%)		Number of patients on hypoglycaemia agents (n,%)		Number of patients on anti-lipid agents (n,%)	
	CKD	No CKD	CKD	No CKD	CKD	No CKD
**FRS <10**	**1 /2(50.0)**	**37/65(56.9)**	**0/0(0.0)**	**18/65(27.7)**	**2/2(100.0)**	**39/65(60.0)**
**FRS 10–20**	**9/14(64.3)**	**164/200(82.0)**	**6/14(42.9)**	**100/200(50.0)**	**8/14(57.1)**	**147/200(73.5)**
**FRS ≥20**	**79/81(97.5)**	**375/393(95.4)**	**45/81(55.6)**	270/392(68.9)*	**56/81(69.1)**	**257/393(65.4)**

FRS = Framingham General CV risk score; CKD = chronic kidney disease

*p = 0.02

Table 6 compares the rate of CVD events in patients with and without CKD according to ethnicity. There is a trend that Indians with CKD have higher CVD events compared to the other two races. However this difference is not significant and the reason for this could be due to the small numbers. Among those without CKD, there was a significant difference in CVD events rate between the races, in that the Indians have the highest number of events. There was significant difference in relationship between CKD and CV events among those Chinese group (p = 0.014).

**Table 6:** Comparison of CVD events and CKD status in different ethinicities (n = 905).

Race	CKD status	CVD event between 1998 and 2007		p-value
		Yes	No	
**Malay**	**CKD**	6(18%)*	**26(82%)**	**0.17**
	**No CKD**	**18 (10.2%)** ^**#**^	**158(89.8%)**	
**Chinese**	**CKD**	11(20.8%)*	**42(79.2%)**	**0.01**
	**No CKD**	**34 (9.5%)** ^**#**^	**325(90.5%)**	
**Indian**	**CKD**	9 (26.5%)*	**25(73.5)**	**0.17**
	**No CKD**	**40 (16.8%)** ^**#**^	**198(83.2%)**	

*p-value = 0.68 for comparison of CVD events in patients with CKD by different ethinicity.

^#^ p-value = 0.04 for comparison of CVD events in patients without CKD by different ethnicity

## Discussion

Our study showed that overall, patients with CKD have higher predicted CVD risk and more CVD events compared to those without CKD. This is consistent with studies which showed that CKD is associated with higher CVD morbidity and mortality.[10, 19–21] Even though CKD is not a variable in the FRS calculation, we have shown that patients with CKD indeed have significantly higher FRS risk score and more CV events compared to those without CKD. Research has showed that CKD patients have higher oxidative stress and atherosclerosis thereby causing higher risk for CVD morbidity and mortality.[22]

Our study also found that CKD status was associated with higher CVD events regardless of ethnicity. Among Chinese patients, the presence of CKD significantly increased the CVD events rate by two-folds while this is not significant in other races. This is consistent with a study done in China which showed that the presence of CKD doubled the CVD risk among Chinese [23]. In India, studies have shown that CKD was associated with higher CVD risk score and CVD events. [1, 24] This same relationship of CKD and CVD risk have been shown among Caucasians population as well. [6, 10, 25] Our study showed that Indians have higher CVD events compared to the other races regardless of the CKD status and this is consistent with a study done in Canada where South Asians like those from India have higher prevalence of CVD compared to Chinese and Caucasians.[26]

The main aim of our study was to examine whether the addition of CKD helps to predict CV risk better, particularly in the medium risk group because it is uncertain as to what should be done for this group of patients. Our study has shown that patients with medium risk but with CKD have the highest number of CV events and this is even higher than the high risk group with CKD. This could be because the medium risk group by virtue of them being of medium risk were not treated as aggressively as those who were at high risk. Hence more CV events occurred in this medium risk group with CKD. This is supported by the treatment profiles where fewer patients who were of medium risk with CKD received treatment compared to those patients of high risk with CKD.

We have shown that including CKD in risk stratification is indeed useful to further identify those who are of medium risk but in actual fact are at higher risk and hence treatment is necessary. Despite being in the medium risk group, those without CKD indeed have risk that is equivalent to those in the low risk group, and hence may not need treatment.

In contrast, other studies factoring CKD into the Framingham equation did not increase the risk predictive ability of FRS. [19, 27–29] This could be due to the studies being done in all patients as an entire group (ie low, medium and high risk). Addition of CKD to low and high risks patients may not change the CV prediction because the absence or presence of other CV risk factors like diabetes, hypertension and dyslipidemia are adequate to predict the CV risk well. Our study also showed that there was no significant difference when comparing the CV event rate between patients with and without CKD in low and high risk groups. However this is not the case in those with medium risk as shown in our study, where the presence of CKD increased the risk score as well the number of events that occurred

The presence of CKD has been shown to increase CV risk and events. Other biological markers such as hsCRP, homocysteine and interleukin-6 have also have been shown to increase CV risk. [30, 31] Imaging parameters like increase in intima-media thickness and left ventricular hypertrophy have also been shown to increase CV risk.[32, 33] However, these tests are expensive, not so readily available and hence not so feasible to be done in most clinical setting. Serum creatinine on the other hand is readily available as it is already part of the routine work-up in someone deemed to have CV risk. Hence no extra expenditure is needed by factoring in CKD to enhance stratifying CV risk further particularly in those with mediun risk where indication for treatment is less certain and clear. Thus taking cognizance of the absence of presence of CKD in those with medium CV risk can help guide clinicians to improve management of their patients in order to reduce CV events in routine daily clinical practice.

### Strengths and limitations

Our study was carried out in a primary care setting where patients’ CV risk profiles are suitable for primary prevention compared to secondary care patients. Another strength is that our study is of a reasonably large sample size and hence is able to show a better prediction of CV risk and relationship between CKD and CVD. Thirdly, our study ascertained CV events over a relatively long interval of 10 years.

As this is a retrospective study, missing data is not unexpected. However the number of missing data was small so is unlikely to affect our findings in any substantial way. Our study may actually underestimate the true CV risk as patients were receiving treatment which would reduce their actual risk. However for the purpose of our study, the under-estimation of the true risk is reasonable as it is still able to show the significant findings as it would be unethical not treating the patients in real clinical practice.

## Conclusion

Presence of CKD causes a higher 10-year CV events rate in those with medium risk determined by the Framingham risk score. Therefore factoring in CKD for those with medium risk helps identify those who are actually at greater risk when treatment may be indicated.
